# A new index for quantifying the ornamentational complexity of animals with shells

**DOI:** 10.1002/ece3.9247

**Published:** 2022-08-26

**Authors:** Luyi Miao, Xu Dai, Hanchen Song, André Ricardo Backes, Haijun Song

**Affiliations:** ^1^ State Key Laboratory of Biogeology & Environmental Geology, School of Earth Sciences China University of Geosciences Wuhan China; ^2^ School of Computer Science Federal University of Uberlândia Uberlândia MG Brazil

**Keywords:** 3D fossil, complexity quantification, echinoderm, mollusk, morphology, ornamentation

## Abstract

Morphological complexity reflects the biological structure of an organism and is closely linked to its associated functions and phylogenetics. In animals with shells, ornamentation is an important characteristic of morphological complexity, and it has various functions. However, because of the variations in type, shape, density, and strength of ornamentation, a universal quantitative measure of morphological complexity for shelled animals is lacking. We propose an ornamentation index (OI) derived from 3D scanning technology and a virtual model for quantifying ornamentation complexity. This index is designed to measure the extent of folding associated with ornamentation, regardless of shape and size. Ornamentation indices were measured for 15 ammonite specimens from the Permian to Cretaceous, 2 modern bivalves, 2 gastropods from the Pliocene to the present, and a modern echinoid. Compared with other measurements, such as the fractal dimension, rugosity, and surface‐volume ratio, the OI displayed superiority in quantifying ornamentational complexity. The present study demonstrates that the OI is suitable for accurately characterizing and quantifying ornamentation complexity, regardless of shape and size. Therefore, the OI is potentially useful for comparing the ornamentational complexity of various organisms and can be exploited to provide further insight into the evolution of conchs. Ultimately, the OI can enhance our understanding of morphological evolution of shelled organisms, for example, whether shell ornaments simplify under ocean acidification or extinction, and how predation pressure is reflected in ornamentation complexity.

## Graphical abstract

We proposed an ornamentation index (OI) derived from 3D scanning technology and a virtual model for quantifying ornamentation complexity. This index is designed to measure the extent of folding associated with ornamentation, regardless of shape and size. The OI can enhance our understanding of fossil morphology evolution and shelled organisms, irrespective of environmental events or ecological system changes.

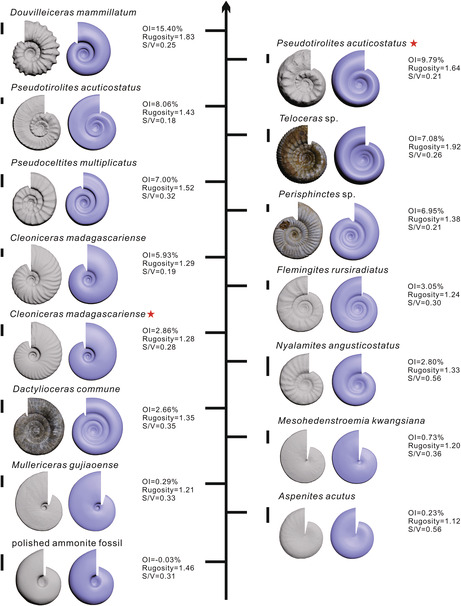

## 1 INTRODUCTION

The morphological characteristics of organisms reflect their biological evolution and have significant implications for their macroevolution, ontogeny, functions, taxonomy, and phylogenetics (Adamowicz et al., 2008; Bonner, 1988; Boyajian & Lutz, 1992; Jones et al., 2019; Reichert et al., 2017; Valentine et al., 1994). Numerous morphometric methods for quantifying morphological evolution in geological history and the interaction of organisms with the environment are available (e.g., morphological disparity mainly reflects changes in morphological diversity and evolutionary trends in shape; Dai et al., 2021; Erwin, 2007; Foote, 1997; Hopkins & Gerber, 2021; Korn et al., 2013; Villier & Korn, 2004; Wills, 2001). Among morphometric indices, morphological complexity indicates the extent of biological structure adaptation because it is significantly associated with form‐function relationships and morphogenetic system‐generation processes. In mollusks, for example, the development and evolution of spine structures may be linked to pressure from shell‐crushing predators (Khanna et al., 2013; Vermeij, 1977) and may also be linked to predation (Herbert et al., 2016; Paine, 1966; Peharda & Morton, 2006; Vermeij, 2001).

The complexity of a clade is usually measured as the number of different part types at a given hierarchical level (McShea & Brandon, 2010; McShea, 2017). In paleontology, the study of fossil complexity is mostly limited to fossil morphology. Therefore, morphological complexity usually refers to the degree of development of an organ or morphological feature that is often used for particular functions in palaeontological research. For example, the morphological complexity of animal shells is mainly influenced by ornamentation (Vörös, 2010; Wu et al., 2019), while in arthropods, morphological complexity is primarily linked to limbs (Adamowicz et al., 2008; McShea, 1996). Consequently, in the past three decades, taxon‐specific approaches for measuring complexity have advanced; these approaches include counting cell types or the number of organs associated with macroevolution (e.g., counting the number of limb types in arthropods; Adamowicz et al., 2008; McShea, 1996; Valentine et al., 1994), cladistic character matrices derived from phylogenetic systems (Deline et al., 2018), 2D and 3D fractal dimensions (e.g., box‐counting, Minkowski–Bouligand dimension; Famoso & Davis, 2016; Fukunaga et al., 2019; Reichert et al., 2017), and biological surface roughness and rugosity (Knauss & Yacobucci, 2014; Martinez et al., 2021; Young et al., 2017). These quantitative and semi‐quantitative methods have been utilized in several studies on biological morphology and its evolution.

**FIGURE 1:**
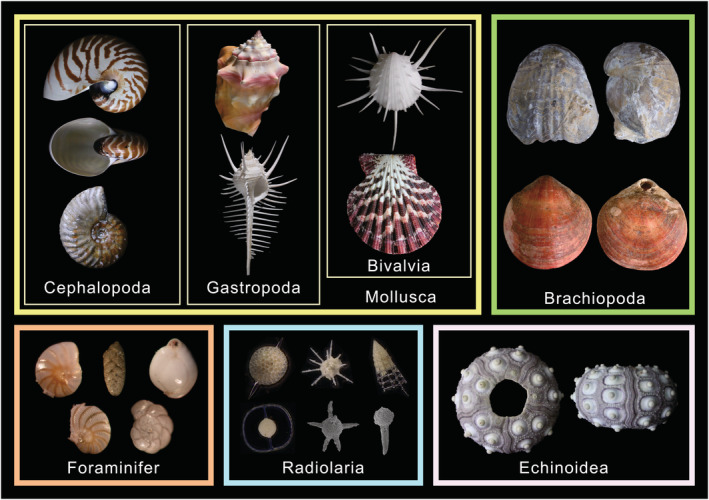
Photographs showing organisms with shells involving varying strength and types of ornamentation.

Nevertheless, a suitable taxon‐free method for measuring the ornamentation complexity of organisms with shells remains elusive. The term “shells,” as used in this paper, refers specifically to carbonate, chitinous, and some siliceous shells of invertebrates (Figure 1). This is partly because of the considerable variety in ornamentation associated with biotic shells. For instance, ammonites exhibit ornaments that include tubercles (nodes, spines, and bullae), parabolic lines, megastriae, varices, constrictions, keels, strigations, and ribs (Hammer & Bucher, 1999; Klug et al., 2015). These ornaments display varying shapes, sizes, densities, and strength combinations depending on the species, which leads to difficulties in contrasting between species. For example, it is difficult to determine which species 1 (with ribs and nodes) and species 2 (with spines) have more developed ornaments, by the different types of characters. In addition, existing methods inadequately exclude body size and shape interference when characterizing ornamentation. For example, in studies regarding the complexity of organisms with irregular shapes, the fractal dimension has been reported to exhibit correlations with rugosity and size (Reichert et al., 2017). Alternatively, geometric morphometrics and theoretical shell models measure geometry directly including ornamentation but not ornament alone (Dai et al., 2021; Liew & Schilthuizen, 2016). In addition, if size overprint cannot be excluded from complexity calculation methods, then the relationship between volume and morphological complexity cannot be further explored.

In recent years, advances in 3D imaging approaches (e.g., X‐ray computed tomography, photogrammetry, laser scanning, gel‐based stereo‐profilometry, atomic force microscopy; Friedman, 2009; Knauss & Yacobucci, 2014; Naglik et al., 2015; Martinez et al., 2021), and virtual modeling (Hammer & Bucher, 2005b; Song, Song, et al., 2021) have improved our understanding of the connection between morphological complexity and ornamentation, as well as functional morphology. However, it is not clear how to quantitatively assess the ornamentational complexity of animals with shells. In this study, the latest 3D scanning technology and artificial modeling were exploited to generate an ornamentation index (OI) that can be employed to adequately quantify ornamental complexity in shelled organisms. The OI was measured for 3D models of conchs of 15 ammonite specimens and 6 other taxa to further illustrate the performance and potential limitations of the OI and the influence of shape and size on OI measurement. Finally, the outlooks on the usages of OI on morphogenesis and morphological evolution were discussed.

## 2 MATERIALS AND METHODS

### 2.1 Shelled animal species and 3D scanning

Eight ammonite specimens, representing seven species from the Late Permian to Early Triassic periods, were obtained from the Yifu Museum at the China University of Geosciences, Wuhan, China (YFMCUG). In addition, four Cretaceous specimens associated with two other species were acquired from the private collection of Xu Dai (one of the authors), while three models of Jurassic ammonites were downloaded from a free 3D fossil database: the Digital Atlas of Ancient Life database (https://www.digitalatlasofancientlife.org). Species with different ornamental types and frequencies, and convex degrees were selected to ensure varying degrees of complexity. Regarding ontogenesis and interspecific variation, we also selected four specimens from two species (*Pseudotirolites acuticostatus* and *Cleoniceras madagascariense*). Therefore, in addition to the well‐preserved fossils, an actual polished ammonite was incorporated as an ideal smooth surface model. In the present study, analysis was limited to well‐preserved specimens, and minor restoration was performed using gypsum when necessary to avoid bias. In addition to ammonites, other main classes of shelled organisms, such as bivalves, gastropods, and urchins, including both fossil and modern species, were examined. Among these organisms, an actual polished bivalve shell of *Codakia tigerina* served as the ideal smooth surface model; data for these specimens and specimen numbers are presented in Appendix S1.

A handheld Arctec Spider 3D scanner with Artec Studio 11 software (Arctec 3D, Luxembourg; resolution up to 0.1 mm and accuracy up to 0.05 mm) was used to scan the specimens. Models were constructed using a 0.2‐mm mesh resolution to minimize loss of ornamentation information to preserve details such as growth lines, while reducing area differences due to fossil material differences. The settings of the “rigid alignment” tool were as follows: fine serial registration, global registration, outlier removal, and smooth fusion (resolution = 0.2 mm, fill holes by radius, max. hole radius: 5 mm). The 3D models were exported as mesh files (Wavefront“.obj”) for subsequent analysis.

### 2.2 Ornamentation index

At present, most of the morphospace models focus only on conch geometry of various invertebrate taxa (Gerber, 2017; Raup, 1962; Raup, 1967), while a few morphospace approaches consider both conch geometry and ornamentations (Dai et al., 2021; Urdy, 2015). However, none of them has quantified the ornamental strength by 3D models.

**FIGURE 2:**
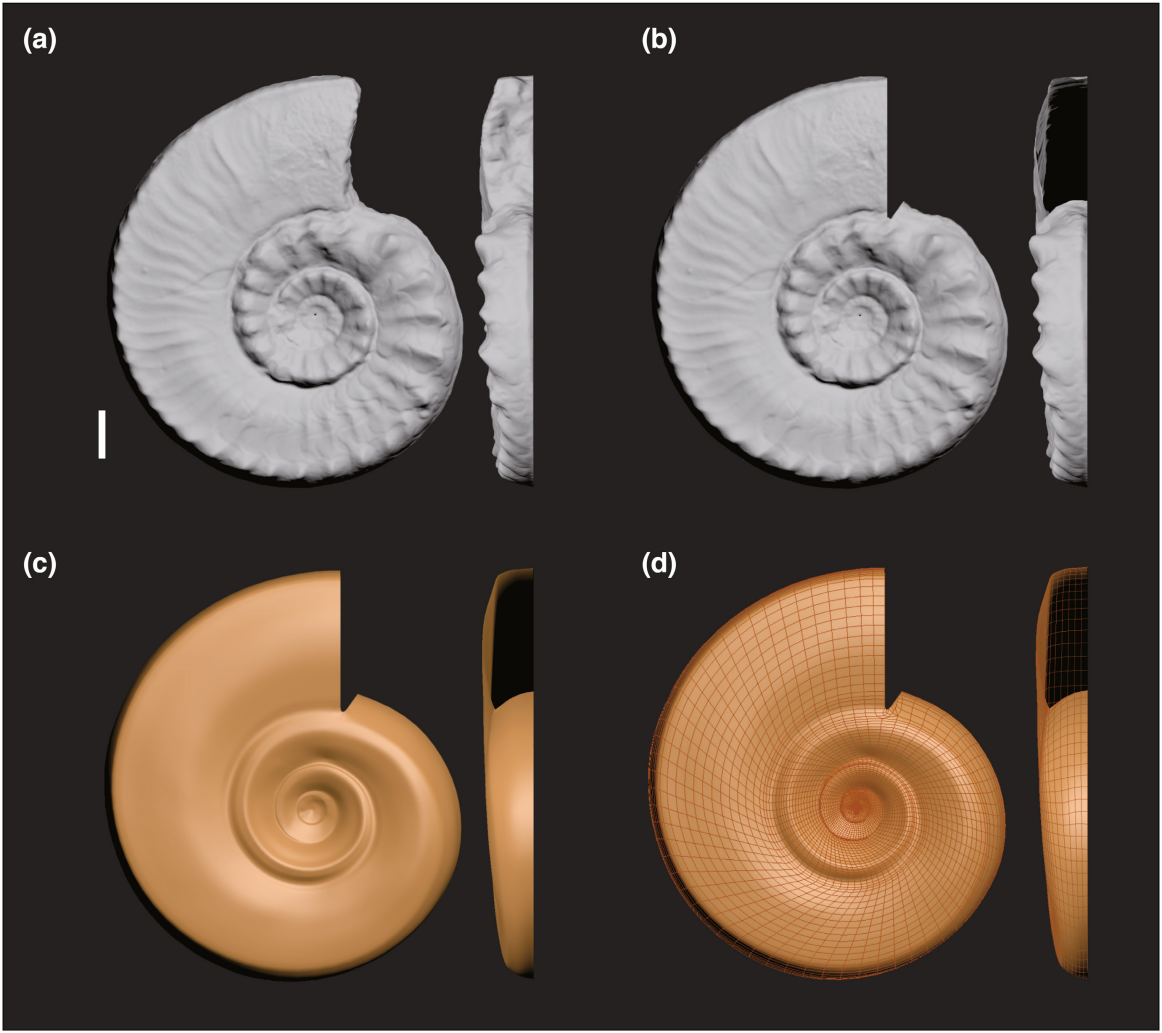
Image of the procedure used to create a model for Pseudotirolites acuticostatus highlighting (a) cutting horizontally along the plane of symmetry to obtain the side exhibiting better preservation, (b) cutting the adoral part to restore the state of an ammonite shell, (c) capturing the curve of the smooth part of shells using 3ds Max to construct an idealized 3D digital model, which preserves the original geometric ratio of the shell conch without ornamentation, and (d) the TurboSmooth process to enhance meshes of the idealized 3D digital model (scale bar = 1 cm).

To quantify the degree of development of conch ornamentations, we minus the whole surface area of a virtual 3D conch model by its manually revised conch model that maintains the original geometry to calculate the area generated by its ornaments (Figure 2). For the obvious ontogenetic variation in geometry (Raup, 1967), manual modeling from the original shell using 3ds Max is the most accurate method.

3D fossil models were imported into 3ds Max 2018 (Autodesk, San Rafael, CA, USA) modeling software. Considering that the studied planispiral ammonites were essentially symmetrical, models were cut horizontally along the plane of symmetry. The portion exhibiting better preservation was retained because of the higher efficiency of a unilateral model for ammonites. All ammonite models displayed closed geometries, and cephalopod shells were characterized by hollow body chambers (Figure 1; modern cephalopod shell) filled during diagenesis. The adoral parts of some specimens also retained several shapes and ornaments. To minimize the uncertainty caused by the rock surface attached to the adoral part, the QuickSlice directive was utilized to remove the adoral surface of the fossil, so that each fossil conch model has an opening chamber (Figure 2b). The same procedure was used to restore the apertures of gastropod fossils.

To generate a smooth model for an ammonite, continuous arcs must be drawn along the lowest parts of the ventrolateral portion, umbilical portion, and ventral centerline. These arcs were then joined to create a surface, adjusted according to the surface smoothness of a fossil that lacks ornamentation. Ultimately, idealized 3D digital models that involved the original geometric ratios of ammonoid conches without ornamentation (Figure 2c,d) were created. In the bivalve model, a fan pattern was drawn along a plane according to the commissure profile at the plane of symmetry between valves. Several radial and concentric coils were then added, and a free‐form deformation select modifier (FFD modifier) was employed to alter the height of the uplift and umbo of the model. Finally, to construct a virtual model for echinoids, a sphere with two open holes at the poles representing the anus and mouth of the urchin was required, and the structure line was adjusted to conform to the shape of the urchin.

The “Measure” tool in 3ds Max 2018 was used to compute the surface areas and volumes of the 3D models. Subsequently, further analyses were performed using parameters linked to the area, and the associated error was verified. To evaluate the deviation between the virtual and corresponding empirical models, the areas for six samples with smooth shells, including four ammonites (Figure 5), a polished bivalve (Figure 4a), and a fossil gastropod (Figure 4d), were investigated.

The OI is intended to reflect the folding caused by ornamentation while excluding the effect of shape and size. Therefore, it involves the extra area created by ornamentation. In the unilateral fossil model, the area is represented by *S*, while *S*′ represents the virtual model; therefore, the OI is defined as follows:

$$\mathrm{OI}=S/S^{\prime }-1$$

### 2.3 Evaluation of the OI relative to other surface complexity quantification methods

To test whether the OI is suitable for accurately quantifying ornamental complexity, the OI data were compared with those for the *S*/*V* ratio, rugosity, and multi‐scale fractal dimension (MFD), as shown in Table 1.

**TABLE 1:** Overview of three dimensional morphological methods for applicable research objects and their characteristics

3D approach	Formula	Analytic target	Whether affected by shape	Whether affected by size
*S*/*V*	*S*/*V*	Surface area for the same volume of irregular forms (Lewis Jr, 1976; Reichert et al., 2016)	Yes	Yes
Rugosity	*S*/Sp	Roughness per unit area (Knauss & Yacobucci, 2014; Young et al., 2017)	Yes	No
MFD	$3-\lim_{r\to 0}\frac{\log\left[V\left(r\right)\right]}{\log\left(r\right)}$	Complexity of irregular shape (Backes et al., 2010; Reichert et al., 2017)	Yes	No
OI	*S*/*S*'‐1	Ornamental strength of whole shell	No	No

Abbreviations: *r*, radius of the expanding sphere; *S*, area; *S*', the area of the smooth virtual model; Sp, the projected planar area; *V*(*r*), the influence volume of all spheres after the dilation process; *V*, volume.

The *S*/*V* ratio was calculated using the area and the volume (Lewis Jr, 1976; Reichert et al., 2016), while surface rugosity was based on the ratio of the 3D surface area to the projected planar area (Young et al., 2017). For ammonites, rugosity is the area of the unilateral fossil divided by the area of the plane of symmetry; therefore, the MFD was estimated based on the Bouligand–Minkowski equation, which is expressed as follows:

$$D=3-\lim_{r\to 0}\frac{\log\left[V\left(r\right)\right]}{\log\left(r\right)}$$

The Bouligand–Minkowski method is based on the influence volume of an object computed from its dilation (Backes et al., 2010). All vertices of a 3D model are the center of dilation spheres in which the dilation radius is variable *r*. *V*(*r*) is the influence volume of all spheres after the dilation process, while *r* represents an absolute value of 1, and this is the resolution of the mesh in the 3D model (0.2 mm in the present study; Florindo et al., 2015; Vorsatz et al., 2021). The fractal dimension (D) was calculated for every model with dilation radii of 3–20 (Reichert et al., 2017; Vorsatz et al., 2021), while log[*V*(*r*)] was estimated using the 3D Bouligand–Minkowski toolbox, which is available at no cost at https://www.facom.ufu.br/~backes/mink3d.html. Afterward, the approximation of a derivative function utilizing finite differences was computed. This study only computed the MFD of 12 ammonite specimens scanned using the Artec Spider 3D handheld scanner with the same settings since the calculated MFD results were related to the quality and resolution of the 3D models used (Reichert et al., 2017).

## 3 RESULTS

### 3.1 Ornamentation index, *S*/*V* ratio, rugosity, and MFD of ammonites

**FIGURE 3:**
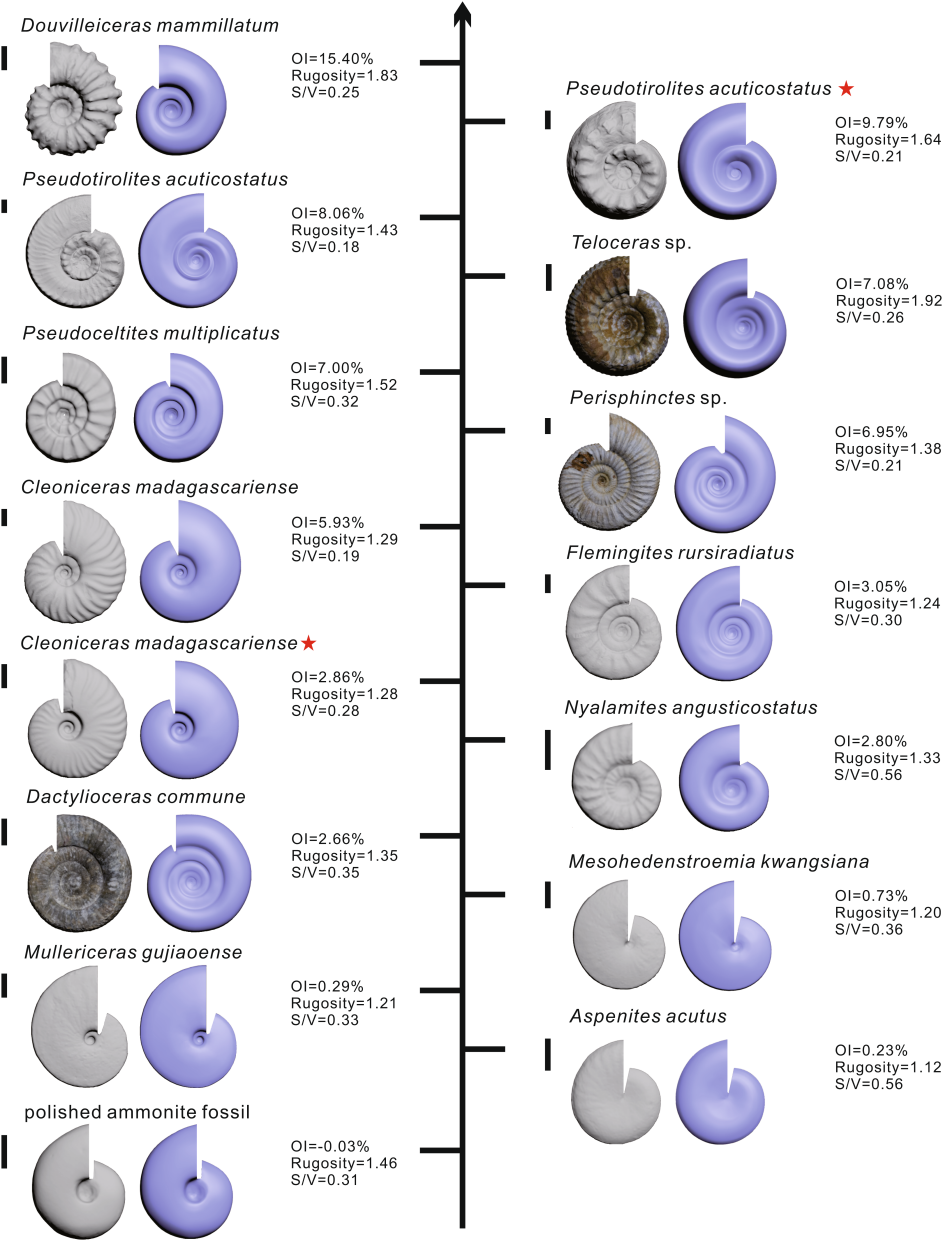
Illustration of the quantification of the morphological complexity for ammonite species including Douvilleiceras mammillatum, Pseudotirolites acuticostatus, Pseudoceltites multiplicatus, Cleoniceras madagascariense, Flemingites rursiradiatus, Nyalamites angusticostatus, Nyalamites angusticostatus, Mullericeras gujiaoense, Mesohedenstroemia kwangsiana, Dactylioceras commune, Teloceras sp., Perisphinctes sp., and polished ammonite fossil (specimens marked with an asterisk were not fully developed and showed intraspecies differences). Results are presented for the OI, *S*/*V* ratio (mm^2^/mm^3^) and rugosity (scale bars = 1 cm), and based on the OI, the ornamentation complexity varies from high (Douvilleiceras mammillatum) to low (polished ammonite fossil).

The trends associated with the OI, *S*/*V* ratio, rugosity, and MFD data for the 15 ammonite specimens are inconsistent (Figure 3 and Appendix S2), which is not surprising as these indexes have different dependencies on conch size, ornamentation type, etc. The OIs, *S*/*V* ratios, and rugosity values vary correspondingly between −0.03–15.40%, 0.18–0.56, and 1.12–1.92, respectively. The highest OI (15.40%) was obtained from *Douvilleiceras mammillatum*, while the maximum *S*/*V* ratio (0.56) was from the smallest *Nyalamites angusticostatus* and *Aspenites acutus*, and the highest rugosity value (1.92) was associated with *Teleoceras* sp. Congruently, the lowest OI, *S*/*V* ratio, and rugosity values were obtained from the polished fossil ammonite (−0.03%), *Pseudotirolites acuticostatus* (0.18), and *Aspenites acutus* (1.12), respectively.

The MFD data exhibit minor differences, and among the 12 specimens examined, the highest value was obtained from the smooth *Mesohedenstroemia kwangsiana* (Appendix S2). Therefore, quantification using the fractal dimension is dependent on a holistic shape and may be limited due to the planispiral structure of ammonites, and thus, it inadequately reflects ornamentation differences.

### 3.2 Ornamentation index of mollusks and echinoids

**FIGURE 4:**
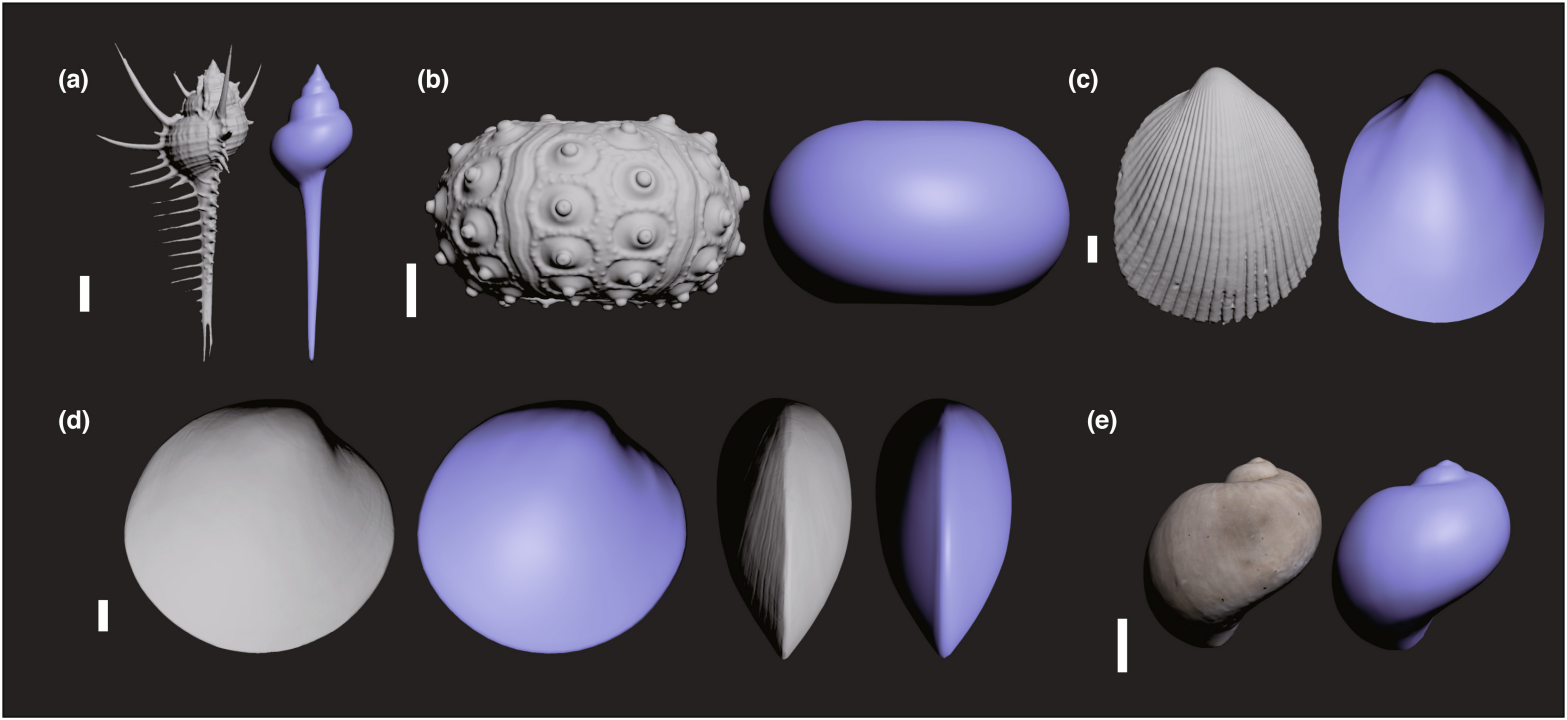
Image highlighting the diversity of the ornamentation index for shelled organisms. (a) a modern sea snail shell (Murex pecten), OI = 61.81%, (b) modern sea urchin shell (Cidaris cidaris), OI = 16.09%, (c) a modern sea bivalve shell (Trachycardium enode), OI = 12.96%, (d) polished bivalves (Codakia tigerina), OI = 0.42%, (e) a gastropod fossil specimen (Naticarius plicatella) obtained from the Digital Atlas of Ancient Life database (https://www.digitalatlasofancientlife.org), OI = 0.74%. Scale bar = 1 cm.

The OIs determined for fossil and modern gastropods, bivalves, and urchins (Figure 4) ranged from 0.42 to 61.81%. The highest OI (61.81%) was associated with *Murex pecten*, which is characterized by three columns of long and thick spines. However, the gastropod specimen *Naticarius plicatella* was smooth and thus produced a low OI of 0.74%. Among the bivalves, only *Trachycardium enode*, which has thick radial ribs, produced an OI value of 12.96%, while the polished shells of *Codakia tigerina* yielded an OI value of 0.42% (Figure 4a). The shell of the modern urchin, *Cidaris*, generated an OI value of 16.09%.

## 4 DISCUSSION

### 4.1 Test effect of shape and size on OI, *S*/*V* ratio, rugosity

**FIGURE 5:**
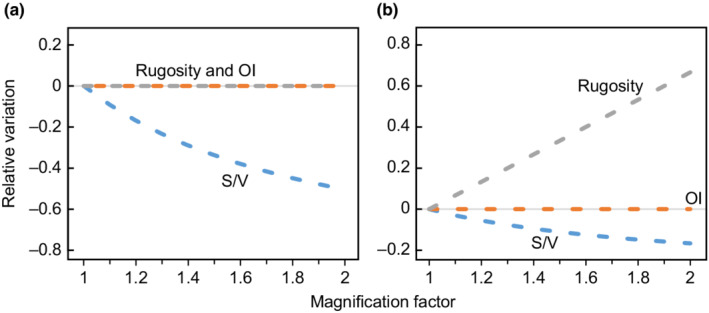
Plot showing the variation of the surface–volume (*S/V*) ratio (mm^2^/mm^3^), ornamentation index (OI), and the rugosity of a model as the size and shape change. (a) the 3D component of the model is magnified by two, (b) the height of the ideal model is doubled.

To evaluate the effect of conch size on the OI, an ammonite specimen (*Douvilleiceras mammillatum*) was scaled, and the variation in the OI, *S*/*V* ratio, and rugosity values for its virtual model at different magnifications was obtained (Figure 5a). With scaling, the area and volume are scaled accordingly using square and cubic multiples of the length. Thus, as model magnification varies, the OI and rugosity remain constant, while the *S*/*V* ratio decreases significantly.

**FIGURE 6:**
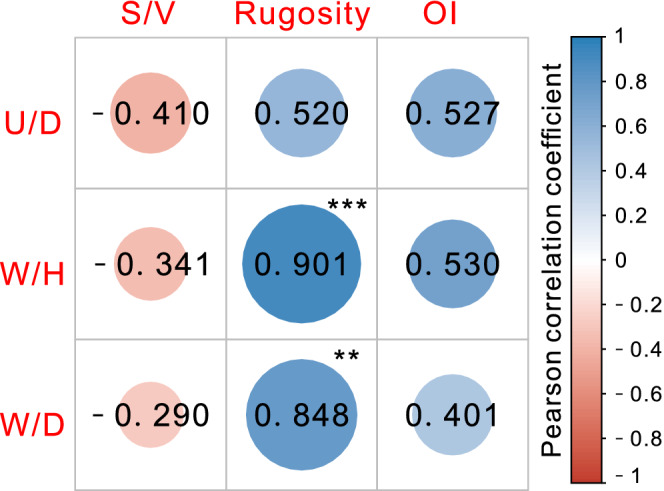
Plot showing the relationships between shape and complexity metrics including the associated Pearson's correlations coefficient. The strengths of the correlations are differentiated based on the colors and sizes of the circles (*p < .05, **p < .01, ***p < .001). The parameters U/D = umbilical diameter/diameter, W/H = width/height, W/D = width/diameter, *S*/*V* = surface–volume ratio (mm^2^/mm^3^) and OI = ornamentation index.

The planispiral shape of ammonites is correlated with the involution level, expansion rate, and thickness, and thus, it partially influences the quantification of surface complexity. Therefore, to assess the impact of shape, the variation in each parameter with changes in ideal model height was simulated (Figure 5b). Evidently, as the height increases, the size of the body also increases; thus, the *S*/*V* ratio decreases, while rugosity increases alongside the area and the constant projected area while the OI remains constant. The correlations between shape and various indicators clearly demonstrate that rugosity is influenced by shape. The increase in surface‐to‐planimetric (3D to 2D) area ratio caused by conchs width has been reported in previous 3D studies (Knauss & Yacobucci, 2014). The shape was further assessed using the umbilical diameter/diameter (U/D), width/height (W/H), and width/diameter (W/D), and correlations between complexity and shape metrics were established using a pairwise plot based on Pearson's correlations (Figure 6). The rugosity displays strong positive correlations with whorl width (W/H) and the total thickness (W/D). Therefore, these results demonstrate that rugosity is influenced by whorl and umbilical widths in addition to ornamentation intensity.

Fractal dimension is also influenced by shape and size. Fractal dimension describes only one aspect of complexity (i.e., the inference of irregularity patterns based on space occupation and self‐similarity); therefore, it neglects certain characteristics (Reichert et al., 2017). Therefore, when comparing associations between space occupation and ornamentation in ammonites, the fractal dimension is dominantly controlled by space occupation related to shape.

The main advantage of the OI is that it enables the accurate distinction of strength of ornamentation without any influence from shape and size. Furthermore, the unrestricted resolution of the model is another advantage of the OI relative to the MFD (Reichert et al., 2017). Therefore, compared with other 3D shape analysis measurements (e.g., rugosity, *S*/*V* ratio, and MFD) that require tests for applicability to a taxon, the OI directly measures the ornamental complexity. An example of the applicability of OI for shelled organisms is shown in Figure 4 and Table 1.

### 4.2 Feasibility and prerequisite of OI

Although the results of the present study demonstrate the potential of the OI for analyzing the ornamental strength of shelled organisms, the approach involves multiple conditions and limitations. The process involved in determining the OI is significantly slower than other methods. In addition, the time required for manual modeling is more than software computation. However, despite the feasibility of artificial modeling using empirical formulae (Hammer & Bucher, 2005b), individual growth varies among organisms. Thus, exclusively using mathematical modeling makes it difficult to restore biological models, and manual modeling is more accurate. Further research could focus on automatized calculations that directly identify the geometric forms in 3D and form smooth models.

Modeling using software (e.g., 3ds Max) can introduce errors between virtual modeling and material objects, whereas the error associated with artificial modeling is negligible. The OIs for all smooth shells were <1% (Figure 3), indicating that virtual modeling was controlled within a low margin of error. These errors may be attributed to artificial friction, weak ornamentation, growth line variations, rock surface differences, and variable damage. In addition, well‐preserved and mature shells are required for modeling. Although some damage can be repaired, human errors can occur during gesso repair.

Mature organisms are required because ornamentation in many species is characterized by growth allometry. For example, the ribs and nodes of *Pseudotirolites acuticostatus* gradually decrease with increasing maturity. Furthermore, the OI for organisms such as *Cleoniceras madagascariense* exhibits intraspecies variation, with a larger value approximately twice the lower value (Figure 3). Therefore, in studies employing the OI to assess interspecies variation, the specimens utilized should reflect the range of the associated differences.

### 4.3 Outlook

The OI proposed in the present study is a quantitative method for evaluating the conchs of different animals, such as ammonites, bivalves, gastropods, echinoderms, and brachiopods. Existing studies on the morphological evolution of shelled animals rely on counting ornament types and characterizing their shapes (Dai et al., 2021; Keller & Abramovich, 2009; Vörös, 2010; Ward, 1981), but these are inadequate for quantitatively measuring the strength of ornamentation. Thus, OI can help understand the macroevolution of the morphology of diverse shelled animals, primarily for evolutionary comparisons within a clade, but also for comparisons among clades.

Cope's rule proposes that animal lineages evolve toward larger body sizes over time (Heim et al., 2015; Stanley, 1973). As a by‐product of Cope's rule, the phenomenon of some structures growing faster than body size (i.e., ornament size shows positive allometry) was confirmed to be present on the sutures and geometry of ammonites (Guex, 2003; Monnet et al., 2011; Raia et al., 2015). This rule has not yet been verified on ornament of shelled‐invertebrate, while sutures cannot be classified as external ornamentation of ammonites. Ornaments of some ammonites do not become stronger during an individual's ontogeny, as seen in some species (i.e., *Pseudotirolites acuticostatus*). Therefore, whether there is a law of faster growth of morphological complexity than body size in ammonites needs to be verified. Ornamentation index can provide a possibility to discuss whether Cope's rule applies to morphological complexity of shelled animals. Buckman's law of covariation, which discusses the relationship of ribbing and shell geometry of ammonites (Guex et al., 2003; Hammer & Bucher, 2005a; Monnet et al., 2015; Moulton et al., 2015; Westermann, 1966; Yacobucci, 2004), could also be quantified through OI. The link between the complexity of suture lines and ornaments was inferred by the Buckman's law (Johnson et al., 2021; Lemanis et al., 2016), could be quantitatively assessed by the OI.

Based on the level of macroevolution, the OI can provide further insight into the relationships between biotic and abiotic changes and their associated biological adaptations. According to previous studies, the functions of ornaments highlight the primary responses of shelled animals to environmental disturbances and predatory pressure; for example, ornament development has been implicated in the burrowing efficiency of infaunal bivalves (Stanley, 1981) and helped deter drilling predation on bivalves (Klompmaker & Kelley, 2015). It has been argued that gastropods and ammonoids developed higher resistance to predatory crushing through strength of ornamentation during the Mesozoic marine revolution (Vermeij, 1977; Ward, 1981), which involved a predator–prey interaction. However, previous studies on the Mesozoic marine revolution lack quantitative methods to measure the ornamental strength. The OI will help elucidate the characteristics and evolution of ornaments associated with the Mesozoic marine revolution, for example, the structures of shelled animals before and after the revolution.

The morphological complexity of shells reflects the difficulty associated with their generation by organisms. By quantifying the OI, the evolution of shelled organism ornaments in response to environmental changes can be alternatively assessed. For instance, ocean acidification could alter shell morphology and reduce ornamentation in gastropods and foraminifera (Harvey et al., 2016; Khanna et al., 2013), while variations in the calcification rate are linked to a changing temperature gradient that could impact ornamentation in brachiopods (Wu et al., 2019). During geological history, several major climatic and environmental events have occurred, including global warming and cooling events (Scotese et al., 2021), oceanic anoxic events (Song et al., 2017), and ocean acidification events (Hönisch et al., 2012). These events have led not only to mass extinctions (Song, Kemp, et al., 2021) but also to remarkable changes in body size (Lilliput effect) and morphology (Calosi et al., 2019; Dai et al., 2021; Feng et al., 2020; Harries & Knorr, 2009; Keller & Abramovich, 2009; MacLeod et al., 2000; Nätscher et al., 2021; Twitchett, 2007). A semi‐quantitative study on ammonoid morphology across the Permian–Triassic mass extinction revealed that ammonoid conch exhibited an ornamental simplification (Dai et al., 2021). Further implication of the OI method will help to elucidate the detailed extent of this ammonite ornamental simplification event, which might have been caused by the increased cost of shell building under ocean acidification, anoxia, and high temperature conditions. The accuracy and efficiency of the OI method could be further enhanced by increasing the analysis of more 3D fossil models in future studies.

In addition to the morphological feedback linked to environmental changes, studies on morphological complexity can improve our understanding of the impact of organisms with varying complexities on other communities. For example, the evenness of the epizoic micro‐community was shown to be influenced by gastropod shell complexity (D'alelio et al., 2010).

## AUTHOR CONTRIBUTIONS

**Luyi Miao:** Conceptualization (equal); data curation (equal); formal analysis (equal); methodology (equal); visualization (equal); writing – original draft (equal); writing – review and editing (equal). **Xu Dai:** Data curation (equal); resources (equal); writing – original draft (equal). **Hanchen Song:** Data curation (equal); formal analysis (equal). **André Ricardo Backes:** Data curation (supporting); formal analysis (equal). **Haijun Song:** Conceptualization (lead); methodology (equal); writing – original draft (equal); writing – review and editing (equal).

### OPEN RESEARCH BADGES

This article has earned Open Data and Open Materials badges. Data and materials are available at https://doi.org/10.6084/m9.figshare.17643446.

## Supporting information


Appendix S1

Appendix S2

## Data Availability

All data used for this research are deposited in figshare https://doi.org/10.6084/m9.figshare.17643446 (Miao et al., 2021).
