# Supplementary material for: A new index for quantifying the ornamentational complexity of animals with shells
Source: Ecol Evol. 2022 Aug 26;12(8):e9247. doi: 10.1002/ece3.9247 (PMC9412138; doi:10.1002/ece3.9247)
Supplement: Supplementary file 2 — Appendix S2 [file ECE3-12-e9247-s001.pdf]

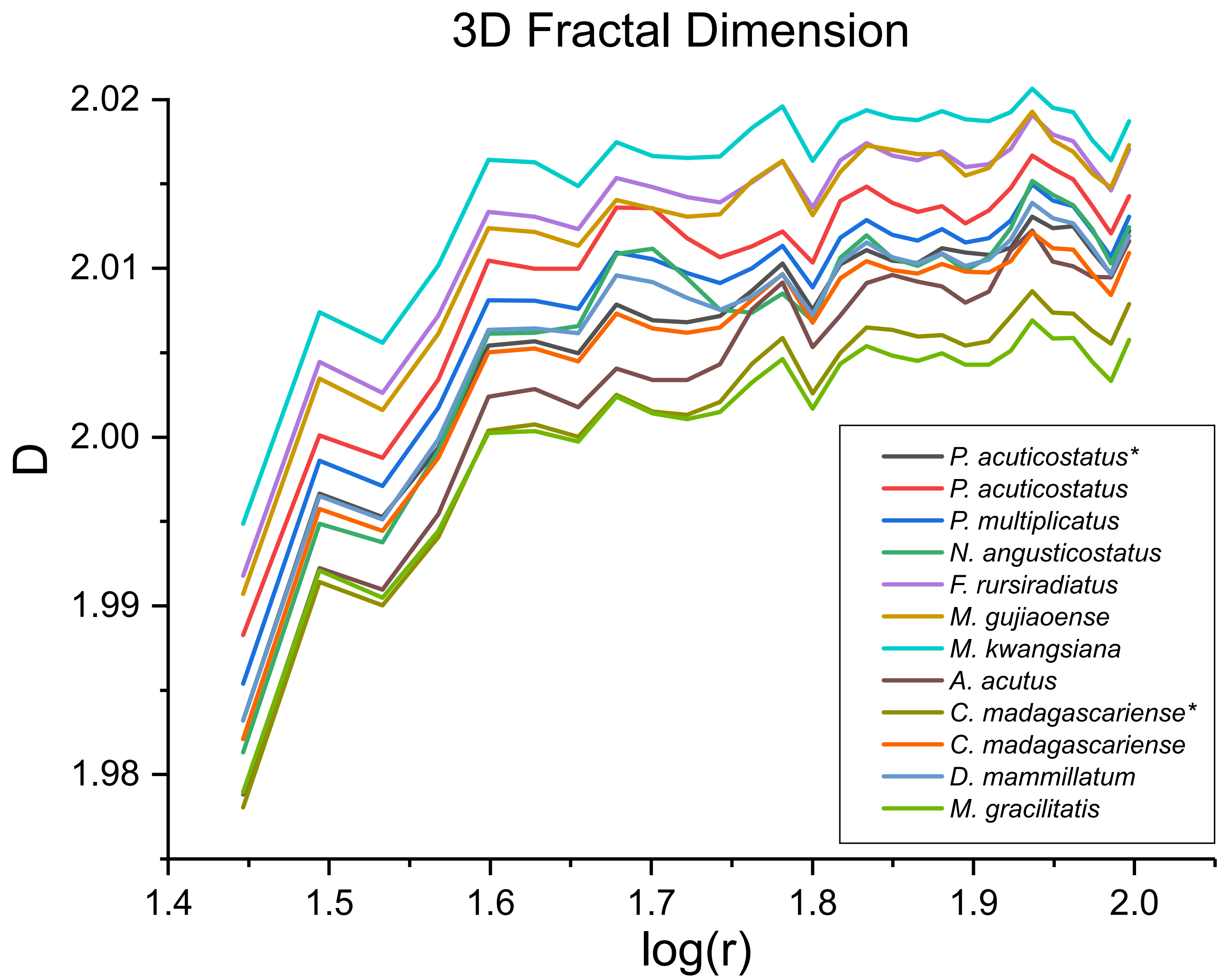

Appendix 2. Multivariate multiscale fractal dimensions are depicted as the fractal dimension D plotted against the dilation radii. Colours designate specimens (specimens marked with an asterisk were not fully developed and showed intraspecies differences).
